# Electrochemical sensing of fentanyl as an anesthesia drug on NiO nanodisks combined with the carbon nanotube-modified electrode

**DOI:** 10.3389/fchem.2022.997662

**Published:** 2022-11-11

**Authors:** Xi Li, Bo Luo, Min Liao, Abdullah Mohamed

**Affiliations:** ^1^ Hospital of Chengdu University of Traditional Chinese Medicine, Chengdu, Sichuan, China; ^2^ Department of Traditional Chinese Medicine, Sichuan Provincial People’s Hospital, University of Electronic Science and Technology of China, Chengdu, Sichuan, China; ^3^ Research Centre, Future University in Egypt, New Cairo, Egypt

**Keywords:** fentanyl, anesthesia drug, hexagonal NiO nanodisks, pencil graphite electrode, voltammetry

## Abstract

Fentanyl was successfully determined in the current effort based on hexagonal NiO nanodisks (HG-NiO-NDs) fabricated by the hydrothermal protocol. The synergism of HG-NiO-NDs with multiwall carbon nanotubes (MWCNTs), large specific surface area, and active material enabled the electrochemical sensor to show potent electrochemical behavior. Admirable performance was found for the fentanyl measurement by the MWCNT and HG-NiO-ND-modified pencil graphite electrode (MWCNT/HG-NiO-ND/PGE). The correlation of oxidation currents with the pH value, concentration, and sweep rate of supporting electrolytes was determined for the optimization of conditions to detect fentanyl. The surfaces of modified and unmodified electrodes were characterized as well. The diffusion-control processes were confirmed on the basis of anodic peak findings. The results also revealed a two-electron transfer process. The linear range was obtained to be 0.01–800.0 μM for the fentanyl concentrations on the developed electrode, with the sensitivity of 0.1044 μA/mM/cm^2^. The limit of detection (S/N = 3) was 6.7 nM. The results indicated the ability of the modified electrode to fabricate non-enzymatic fentanyl sensor applications.

## 1 Introduction

N-(1-Phenyl-4-piperidyl) propionanilide citrate, also called as fentanyl citrate, which is a strong man-made narcotic analgesic, is widely prescribed for analgesia and anesthesia in the intensive care unit and operating room. The effect of 100 μg of fentanyl is estimated to be equivalent to about 10 mg of morphine. Chronic pain can be relieved to some extent with transdermal fentanyl patches (Ebrahimzadeh et al., 2008). Fentanyl is metabolized in the liver after easy passage through plasma and the central nervous system (CNS) (Saffer et al., 2015). The serious complications have been reported for fentanyl, such as serotonin syndrome, respiratory distress, coma, addiction, gastrointestinal conditions, and hypotension. Therefore, it is critical to diagnose this substance as more medically dangerous than heroin (Sohouli et al., 2020). The conventional methods such as radioimmunoassay, surface-enhanced Raman spectroscopy (SERS), and other chromatographic techniques have already been employed to determine fentanyl in biological matrices such as blood and urine (Stiller et al., 1990; Saraji and Boroujeni, 2011; Haddad et al., 2018). Among these, special attention has been paid toward the electrochemical method for the fentanyl determination because of sensitivity, selectivity, low cost, rapidity, and safety (Foroughi et al., 2015; Foroughi and Ranjbar, 2017; Jahani, 2018; Maaref et al., 2018; Farvardin et al., 2020; Foroughi et al., 2021a; Moarefdoust et al., 2021; Santana et al., 2021; Dalkiran and Brett, 2022; Foroughi and Jahani, 2022; Jahani et al., 2022). It is very important to choose the right platform in the electrochemical method, for example, the pencil graphite electrode (PGE) is known as a suitable substrate in electrochemical applications with unique properties such as broad potential range and low background current (Antherjanam and Saraswathyamma, 2022). The electron transfer is slow on the surface of this green platform. This bottleneck can be bypassed by modifying the electrode surface with different strategies. Chemically modified electrodes (CMEs) have been recently introduced for different electroanalysis and (bio)sensors (Salajegheh et al., 2019; Al-Enizi et al., 2020a; Ubaidullah et al., 2020a; Vakili Fathabadi et al., 2020; Foroughi et al., 2021b; Vignesh et al., 2022).

Due to their high electrical conductivity, chemically modifiable surface area, large surface area, chemical stability, high mechanical strength, and high surface-to-volume ratio, carbon nanotubes (CNTs) are also attractive for electroanalysis. They are able to enable the oxidation of the analyte (You et al., 2021; Zhou et al., 2021). Carbon nanotubes containing metal oxides are expected to form a hybrid nanostructure for electrochemical sensors.

Metal oxide nanocrystals have distinct geometric shapes and sizes due to attractive size/shape/surface structure-dependent attributes and high potential as basic building blocks for nanoscale electronic and photonic equipment. Nickel oxide (NiO) is a p-type semiconductor and has a bandgap as broad as 3.6–4.0 eV (Yang et al., 2008; Ahmad et al., 2019; Fathi et al., 2020; Khand et al., 2021; Haunsbhavi et al., 2022). It has various applications such as catalysts, electrode materials for lithium ion batteries, electrochromic films, electrochemical supercapacitors, sensors, photovoltaic tools, and magnetic materials. Promising NiO applications and physicochemical properties of nanoscale materials have led to extensive studies to fabricate NiO nanomaterials with different morphologies (Ichiyanagia et al., 2003; Li et al., 2007; Al-Enizi et al., 2020b; Ubaidullah et al., 2020b).

The current work introduces a sensitive electrochemical sensor (MWCNT/HG-NiO-ND/PGE) with high reproducibility for the determination of fentanyl, based on the fabrication of a new nanostructure of hexagonal NiO nanodisks (HG-NiO-NDs), and then the surface modification of a pencil graphite electrode with the as-fabricated nanostructure. The characteristics of fentanyl were determined using cyclic voltammetry (CV), differential pulse voltammetry (DPV), and chronoamperometry (CHA) techniques.

## 2 Experimental

### 2.1 Chemicals and reagents

The multiwalled carbon nanotubes (MWCNTs) with a diameter of nanotubes (NTs) of OD = 6–13 nm, purity of >98%, and length of 2.5–20 μm were bought from Aldrich. Some local pharmacies were selected to purchase fentanyl tablets. Other required chemicals (belonging to Merck) with a practical grade were included. All solutions were freshly prepared with double distilled water (DDW). To prepare the fentanyl standard solution, an aliquot amount of fentanyl was dissolved in methanol (10 ml), followed by diluting to 50 ml at pH 7 (the final concentration of 1 mM). The lower concentrations were daily obtained by stepwise dilution.

### 2.2. Equipment

A potentiostat (Metrohm 757 VA Computrace, Herisau, Switzerland) with a conical vessel was utilized to perform all voltammetric determinations, and a three-electrode system consisted of one working electrode (pencil graphite electrode (PGE)), one axillary electrode, (Pt) and one reference electrode (SCE). All pH measurements were carried out using a digital Metrohm 710 pH/mV meter. The Rigaku D/MAX-3B powder diffractometer with Cu/Kα radiation at λ = 1.54056 Å was employed to record X-ray diffraction (XRD) with intensities as a function of 2θ. The specimen was scanned at 10–80° (2θ) in 0.02 steps. Surface analysis was performed using images from su35000 field emission scanning electron microscopy (FESEM; (Hitachi; Japan)) and Ametek energy-dispersive X-ray spectroscopy (EDS; Octane Prime; United States).

### 2.3 Construction of hexagonal NiO nanodisks

Porous hexagonal NiO nanodisks were prepared by dissolving nickel nitrate hexahydrate (Ni(NO_3_)_2_.6H_2_O, 1.45 g) and hexamethylenetetramine (1.0 g) in DDW (50 ml) while stirring rigorously for 15 min. The solution pH was adjusted to 13 using some drops of NaOH, followed by stirring vigorously for 60 min. The solution was then sealed and heated up to 150°C for 10 h in a Teflon-lined stainless steel autoclave, followed by cooling down to room temperature. The resultant precipitate was washed with DDW/ethanol and dried at ambient temperature overnight, followed by calcination at 650°C for 3 h. Diverse techniques were applied to characterize the final product.

### 2.4 Construction of the modified electrode

Due to the importance of electrode construction in the electrochemical analysis, the PGE surface was first washed thoroughly with water to remove possible impurities. Then, the MWCNT (1 mg) was dispersed in DDW (1 ml) to prepare the modifier under 15-min sonication. The PGE surface was coated with suspension (6 µL) *via* drop-casting, followed by drying at 50°C in an air oven. Identical methods were adopted to fabricate MWCNT/HG-NiO-ND/PGE with the addition of 6 µL of HG-NiO-ND suspension (1.0 mg HG-NiO-ND + 1.0 ml DDW) on the MWCNT/PGE surface.

### 2.5 Analysis of tablet and serum specimens

Some of the fentanyl powders required to prepare the stock solution (1.0 × 10^−3^ M) were dissolved in DDW under sonication. Aliquots of the clear supernatant of the tablet solution were diluted with phosphate-buffered solution (pH 7) for analysis. Certain amounts of pure drugs were added to the tablet solution under analysis to evaluate the effect of the tablet excipients, the accuracy of the technique.

The serum samples were obtained from a reputable medical laboratory (Pasteur Bam Hospital). The serum sample concentration was adjusted in phosphate-buffered solution before fentanyl analysis.

## 3 Results and discussion

### 3.1 Determination of hexagonal NiO nanodisk characteristics

**FIGURE 1:**
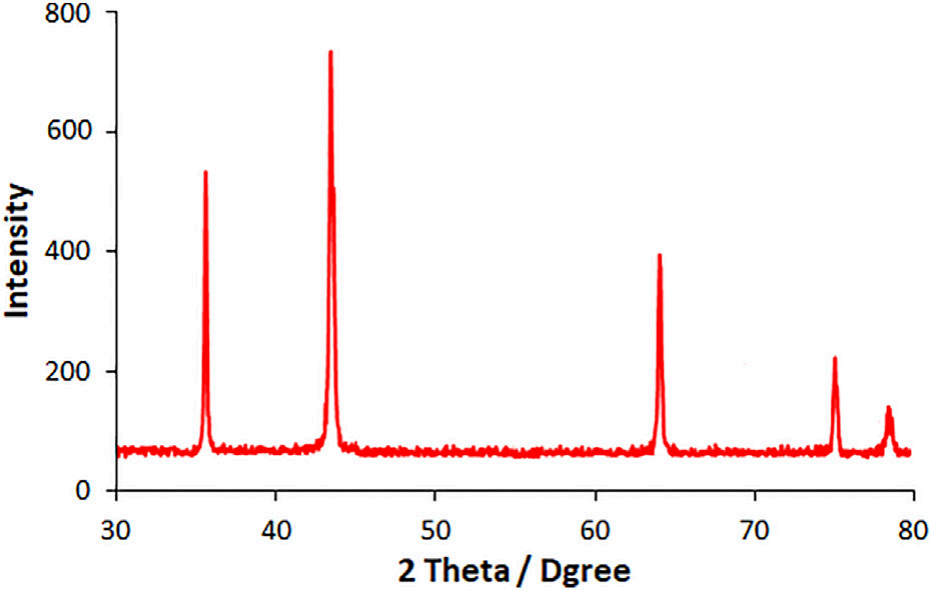
XRD pattern of HG-NiO-NDs.

As-fabricated HG-NiO-NDs were examined for crystallinity, phase, and morphology as follows: the XRD patterns (Figure 1) regarding the crystal structure verified an acceptable crystallinity. The peaks at 35.76°, 43.46°, 63.89°, 74.95°, and 78.68° were related to the planes of (111), (200), (220), (311), and (222), respectively. The XRD data are evidence of a cubic form of NiO (JCPDS No: 78-0643) (Zhou et al., 2018). According to XRD findings, the diffraction peaks corresponded to NiO only. Based on the XRD results, the Scherer equation of D = Kλ/ßcosθ was used to calculate the HG-NiO-ND crystallite size, where λ stands for the used X-ray wavelength (1.541 Å), β for the peak width at half maximum (FWHM), and θ for the Bragg diffraction angle, which was 106.43 nm.

**FIGURE 2:**
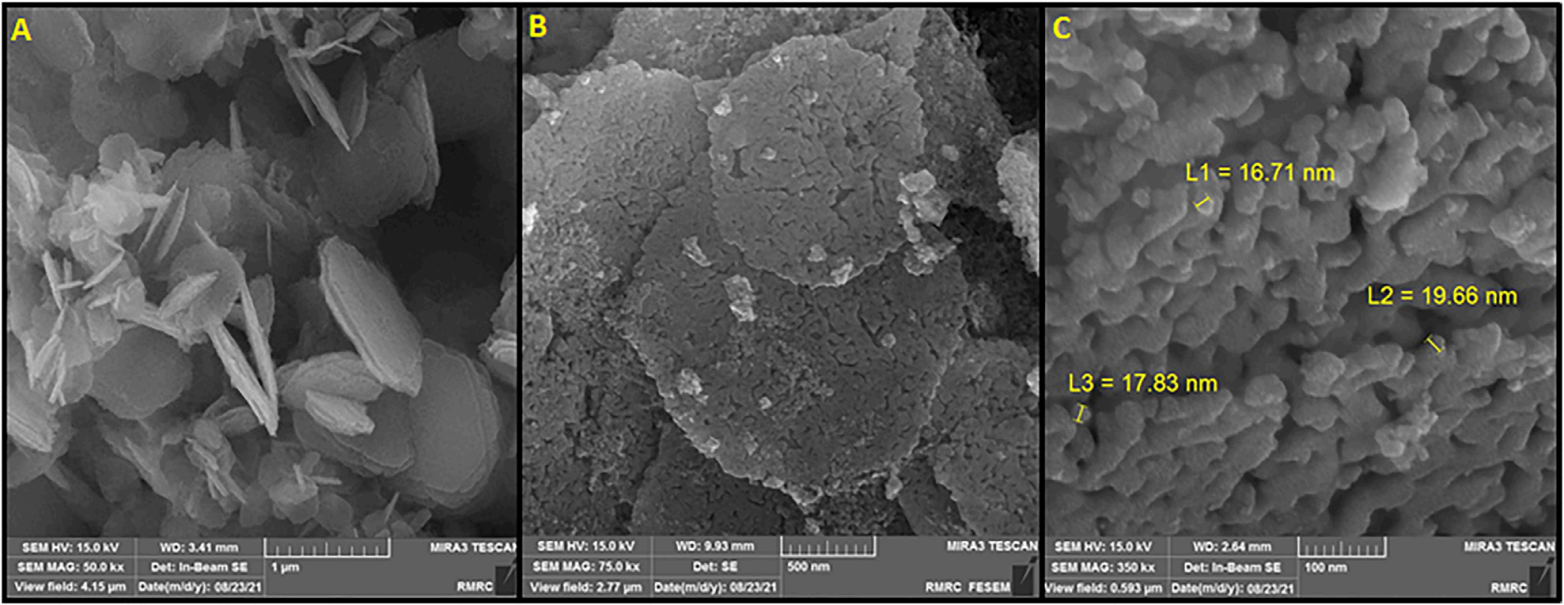
**(A)** and **(B)** FESEM image. **(C)** High-resolution FESEM image of HG-NiO-NDs.

The morphological analysis of HG-NiO-NDs was performed by FE-SEM, the results of which are presented in Figure 2. Interestingly, the prepared HG-NiO-ND possesses hexagonal shape morphologies with high-porous surfaces and irregular pore sizes as shown in Figure 2B. Due to high-porous morphologies, some broken nanodisks are also observed in Figure 2A. It is fascinating to see that most of the nanodisks possess perfect hexagonal shapes with an internal angle of ∼120°; however, there are also some deformed randomly distributed nanodisks. The mean diagonal of nanodisks ranged from 0.4 to 1.5 μm although the micrograph shows some larger nanodisks. The mean typical nanodisk thickness was 10–20 nm, as shown in Figure 2C.

**FIGURE 3:**
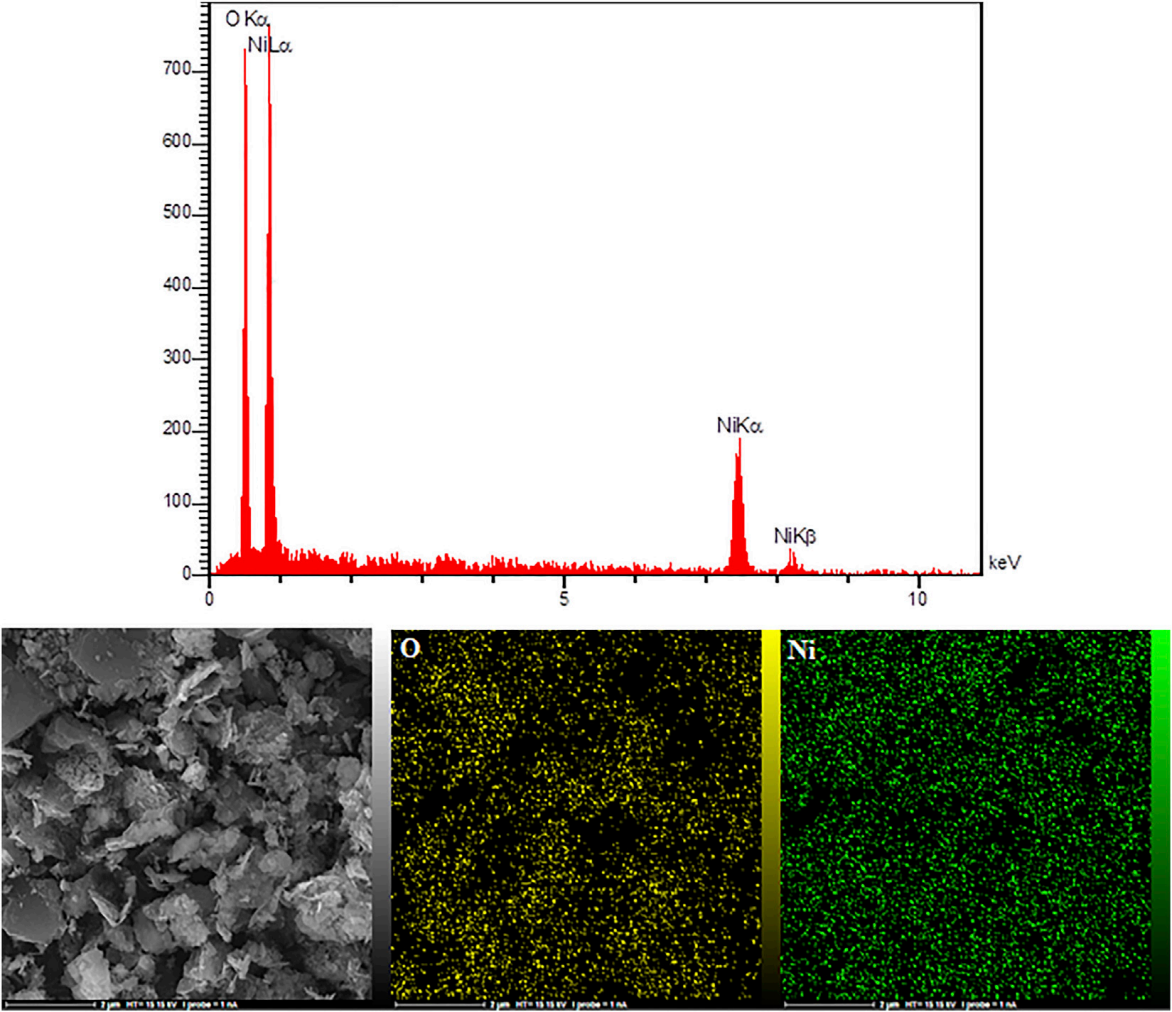
EDX spectra and elemental mapping of HG-NiO-NDs.

Figure 3 illustrates HG-NiO-ND EDS analysis. According to the EDS analysis, the compositions contained only Ni and O, with no impurity. The elemental mapping also confirms the distribution of Ni and O.

**FIGURE 4:**
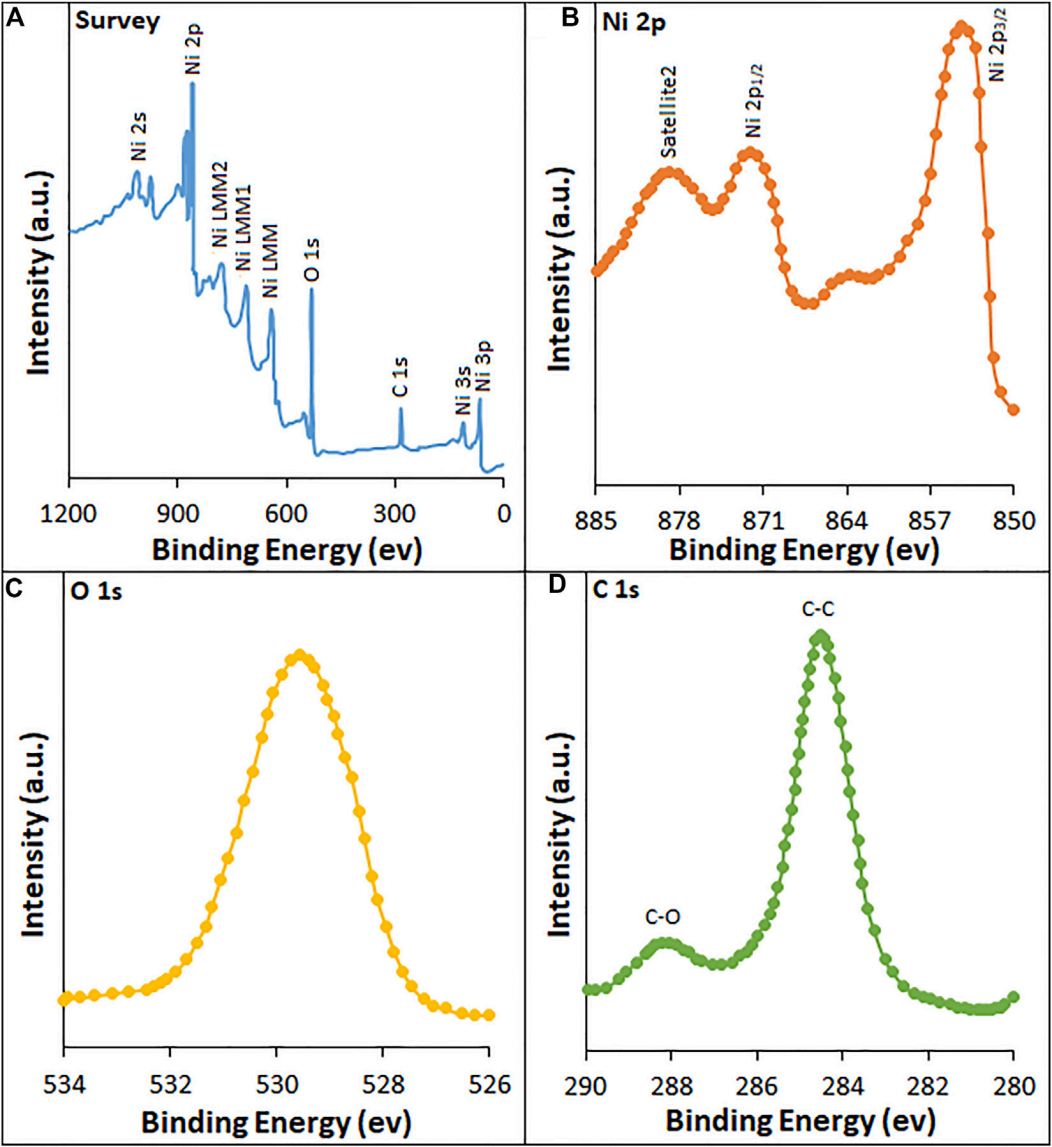
XPS spectra of HG-NiO-NDs: **(A)** survey; high-resolution spectra of **(B)** Ni 2p, **(C)** O 1s, and **(D)** C 1s.

XPS studies (Figure 4) were performed to establish the binding energy and oxidation states of the elements present at the surface of HG-NiO-NDs. The wide XPS survey spectra (Figure 4A) reveal the presence of Ni and O. High-resolution spectra, as shown in Figures 4B–D, typically exhibit 2P_3/2_ which consists of the main peak at ∼853 eV. Similarly, 2P_1/2_ displays two peaks at ∼871 eV and ∼878 eV, corresponding to the main peak and the satellite peak, respectively. The Ni 2P_3/2_ peak at ∼853 eV and O 1s peak at ∼529 eV are from Ni^2+^ and lattice oxygen, respectively, and are incorporated with the Ni–O octahedral bonding of cubic rock salt (Al-Enizi et al., 2021). The carbon peak is originated from surface contamination in the process of handling and storage of the sample (Figure 4D).

### 3.2 Electrochemical behaviors of MWCNT/HG-NiO-ND/PGE

**FIGURE 5:**
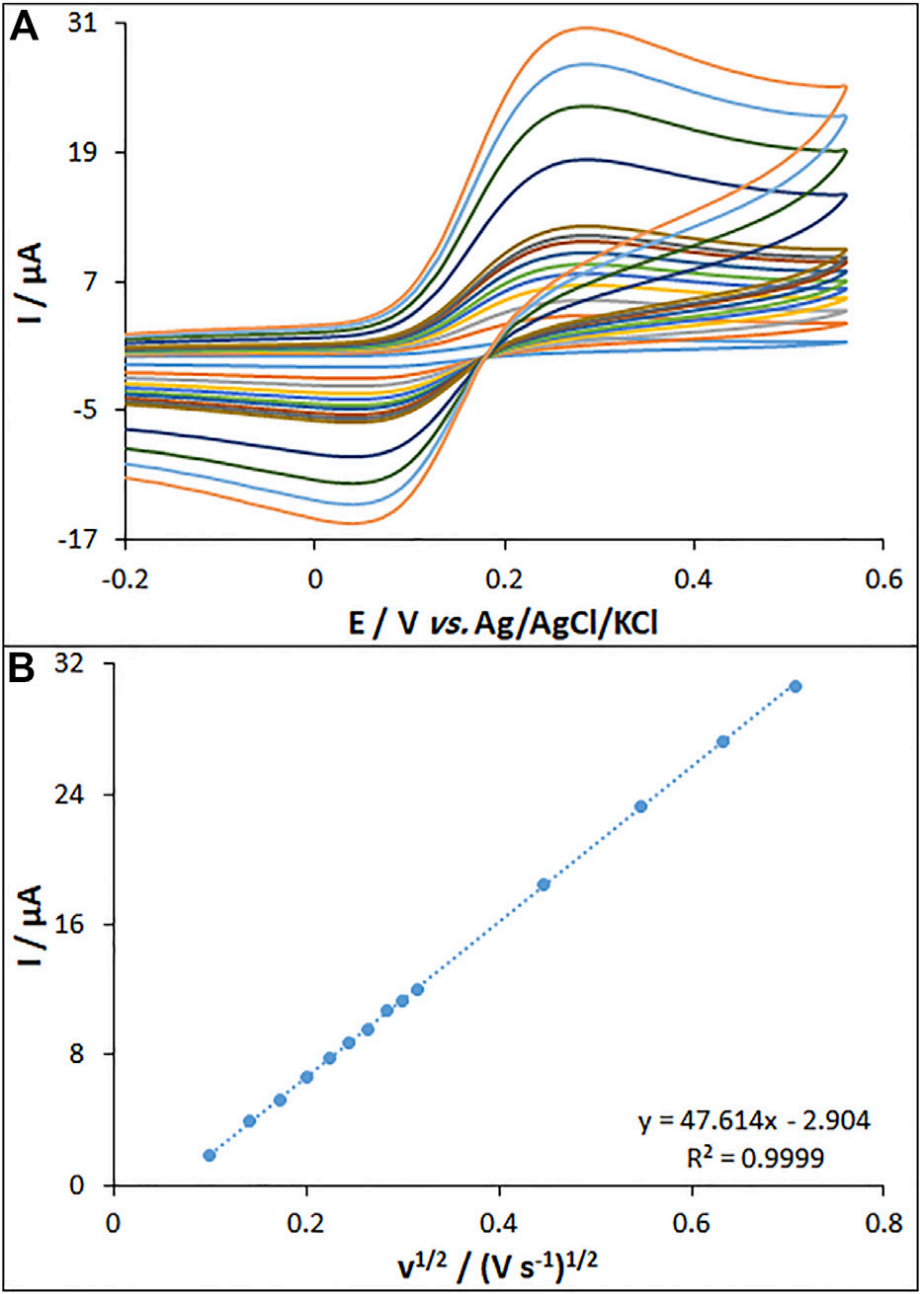
**(A)** CVs of MWCNT/HG-NiO-ND/PGE in the presence of 0.2 mM [Fe(CN)_6_]^3-^ solution in aqueous 0.1 M KCl at various scan rates (from inner to outer curves): 10, 20, 30, 40, 50, 60, 70, 80, 90, 100, 200, 300, 400, and 500 mV/s. **(B)** Plot of peak currents vs. υ^1/2^.

The CV curves were recorded for bare PGE, MWCNT/PGE, and MWCNT/HG-NiO-ND/PGE in the redox probe. Figure 5A shows the redox peaks for MWCNT/HG-NiO-ND/PGE, possessing the peak-to-peak differences (∆Ep = E_anodicpeak_ − E_cathodicpeak_) of 0.19 V. Figure 5B shows the performances of the MWCNT/HG-NiO-ND/PGE according to the Randles–Sevcik equation expressed in Eq. 1 (Bard and Faulkner, 2001):

$$I_{p}=\pm \left(2.69\times 10^{5}\right)n^{3/2}\;AD^{1/2}Cv^{1/2}.$$ (1)

In this equation, the A values are 0.14, 0.19, and 0.32 cm^2^ for the surfaces of bare PGE (BPGE), MWCNT/PGE, and MWCNT/HG-NiO-ND/PGE, respectively.

**FIGURE 6:**
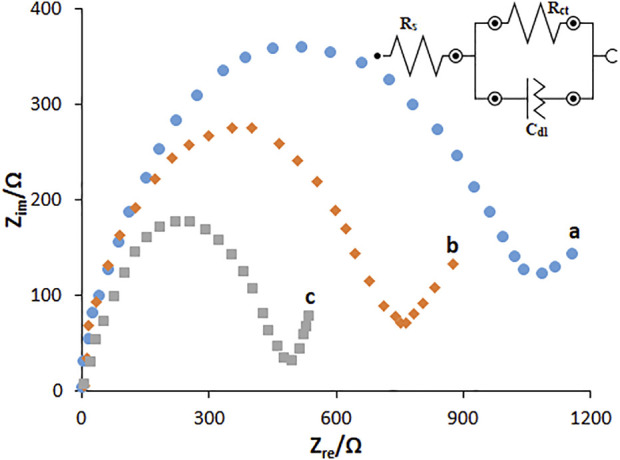
EIS diagrams and the equivalent circuit for 0.1 mM [Fe(CN)_6_]^3-^ solution at **(A)** BPGE, **(B)** MWCNT/PGE, and **(C)** MWCNT/HG-NiO-ND/PGE in aqueous 0.1 M KCl. Frequency ranges from 100 KHz to 0.1 Hz.

The EIS method was used to electrochemically determine the characteristics of HG-NiO-NDs in which the charge-transfer resistance (Rct) shows the electron-transfer kinetics of the redox probe at the electrode interface, confirming the substrate bond on the surface of the modified electrode. Nyquist plots were drawn for the BPGE, MWCNT/PGE, and MWCNT/HG-NiO-ND/PGE in the redox probe (Figure 6). As seen, a large semicircular structure exists for the BPGE at high frequencies, as a high charge-transfer resistance (Rct = 1,194 Ω) related to a low charge and mass transfer rate. MWCNT/PGE and MWCNT/HG-NiO-ND/PGE showed a significant decrease in the Rct values (794 Ω and 483 Ω, respectively), which can be because of capacity of NiO ND and HG-NiO-ND to boost the electron transfer as well as the electrode surface area. The results showed that the resistance is lower after modification with MWCNTs and HG-NiO-NDs, demonstrating that modifiers increase the oxidation peak current of the fentanyl; consequently, it increases the sensitivity.

Based on the EIS, the standard heterogeneous rate constant for electron standard transfer (*k* ^*0*^ *,* cm/s) was calculated to explore the modification of the electrode surface according to Eq. 2 (Bard and Faulkner, 2001):

$$k^{0}=\frac{RT}{F^{2}R_{ct}AC}.$$ (2)

In this equation, *R* stands for the global gas constant (squared with 8.314 J/K/mol), *T* for thermodynamic temperature (298.15 K), *F* for Faraday constant values (96.485 C/mol), *A* for the electrode surface area (cm^2^), and *C* for the concentration of 0.1 mM [Fe(CN)_6_]^3-/4-^.

The *k* ^*0*^ values were computed to be 1.59 × 10^−2^, 1.67 × 10^−2^, and 1.72 × 10^−2^ cm/s for the BPGE, MWCNT/PGE, and MWCNT/HG-NiO-ND/PGE, respectively. The values of K^0^ approach the kinetic potential of the redox couple. Thus, a system with a higher k^0^ value has a longer balance in less time than a system with a lower k^0^ value. Therefore, a greater k^0^ value is obtained for the MWCNT/HG-NiO-ND/PGE sensor than MWCNT/HG-NiO-ND/PGE > MWCNT/PGE > BPGE, indicating faster electron transfer than other electrodes.

### 3.3 The voltammetric activity of fentanyl

**FIGURE 7:**
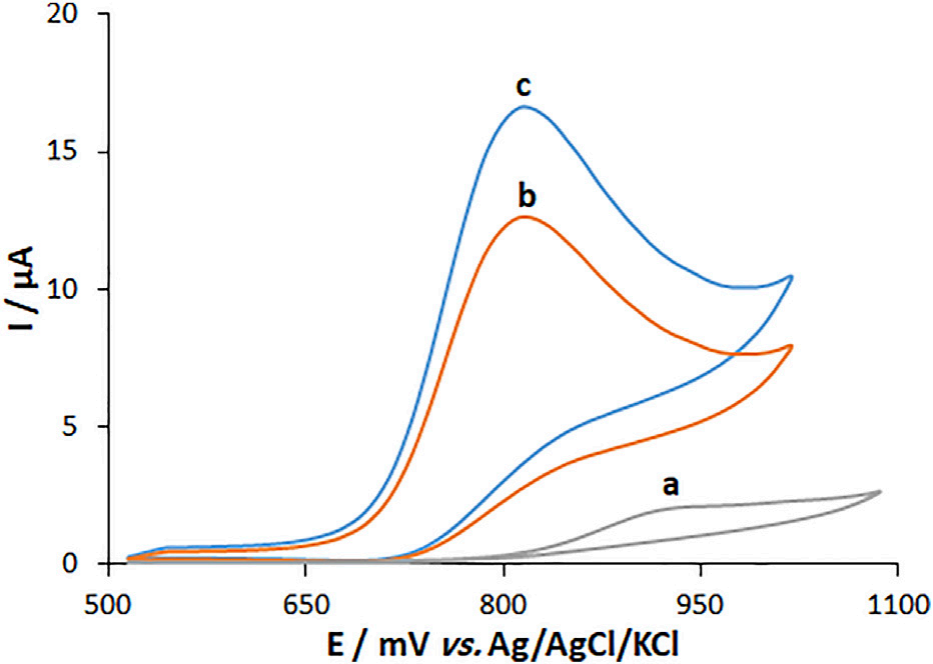
CVs of **(A)** BPGE, **(B)** MWCNT/PEG and **(C)** MWCNT/HG-NiO-ND/PEG in 0.1 M PBS (pH 7.0) containing 160.0 μM fentanyl.

The cyclic voltammetry (CV) method was followed to explore the electrochemical activity of fentanyl (160.0 μM) in 0.1 M PBS at pH 7.0 on the surfaces of MWCNT/HG-NiO-ND/PGE, MWCNT/PGE, and BPGE. The results showed that MWCNT*/*HG-NiO-ND/PGE does not undergo any oxidation reaction in the absence of fentanyl in 0.1 M PBS at a pH of 7.0 (Figure 7 (curve a)). Figure 7 (curve b) shows the fentanyl oxidation peak of 924 mV with an ultra-low peak current on the BPGE. Figure 7 (curve c) shows a distinct peak for fentanyl on the MWCNT/PGE at 821 mV. Figure 7 (curve c) also illustrates six times higher peak current for fentanyl on MWCNT/PGE than on BPGE (curve b) because of the catalytic ability of MWCNTs. Figure 7 (curve d) shows increased peak current due to the augmentation of MWCNTs and HG-NiO-NDs. The electro-catalytic synergism of MWCNTs with HG-NiO-NDs caused the MWCNT/HG-NiO-ND/PGE to improve its catalytic behavior toward fentanyl and was useful for detecting this compound.

### 3.4 The effect of solution pH on fentanyl oxidation

**FIGURE 8:**
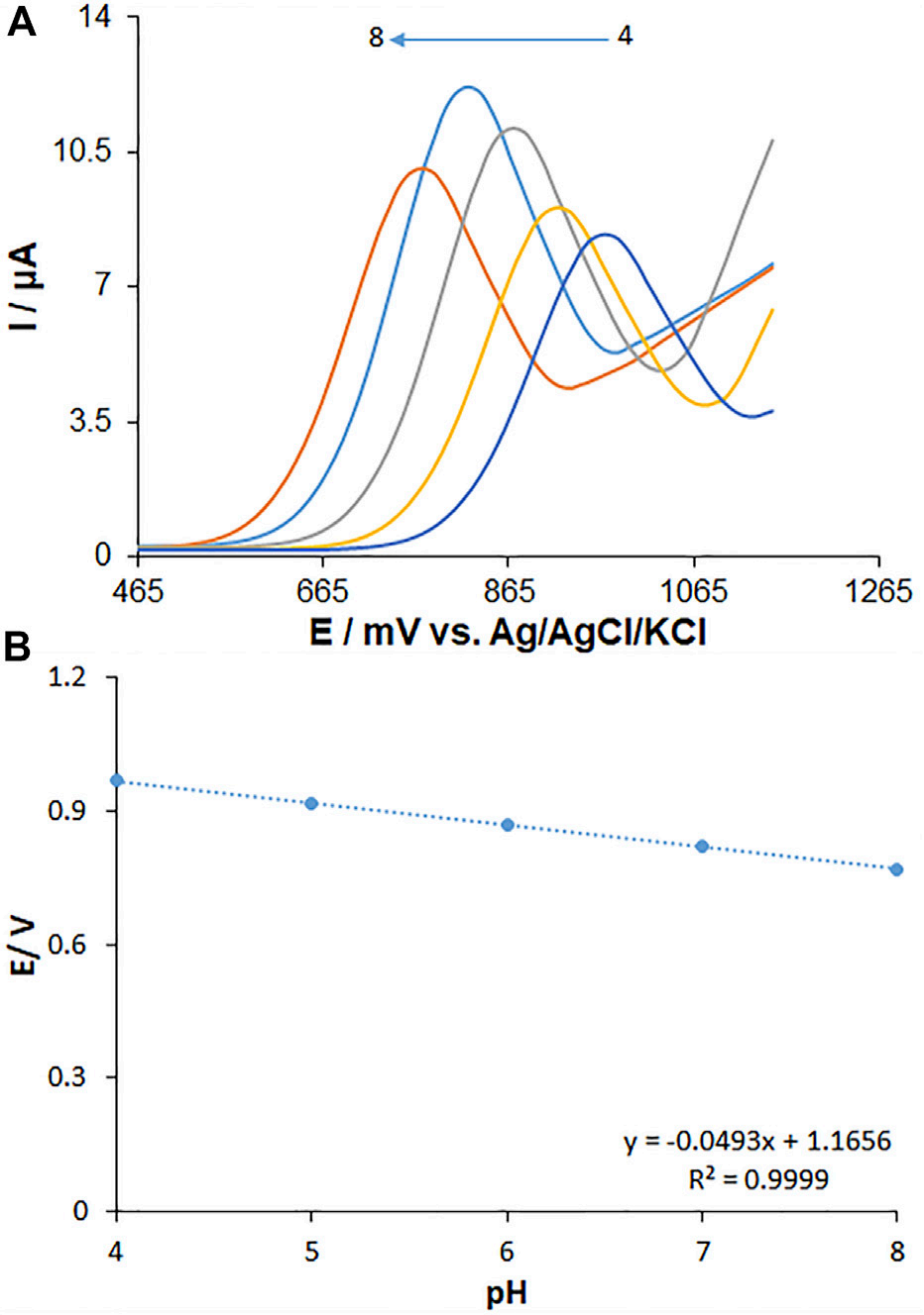
**(A)** Effect of pH on the peak currents for the oxidation of fentanyl (125.0 µM), pH = 4–8. **(B)** Plots of peak potential vs. pH. Scan rate: 50 mV/s.

The solution pH is a pivotal parameter influencing the fentanyl electro-oxidation because of the presence of protons in the electrode reaction. The measurements (Figure 8) were carried out by the CV method regarding the signal of MWCNT/HG-NiO-ND/PGE in 0.1 M buffer solutions at variable pH values from 4 to 8. There was a minor increase in the fentanyl peak currents with increasing solution pH until 7.0 and then decreased. The highest peak current was found at pH 7.0 for fentanyl. Gradually, elevated pH of the solution switched the peak oxidation potential of fentanyl toward less positive values, which shows the presence of protons during the electrode reactions. Since the PBS with pH 7.0 created an optimized reaction for peak current and peak shape and negative shift, the value was selected to be the best (working pH) for next testing. The plot of E_p_ versus pH was drawn for fentanyl at pH 7 (Figure 8B). The E_p_ values of fentanyl had a linear relationship with buffer solution pH as follows:

$$Fentanyl:\;E_{p}\left(V\right)=-0.0493pH+1.1656\;\left(R^{2}=0.9999\right).$$ (3)

**SCHEME 1:**
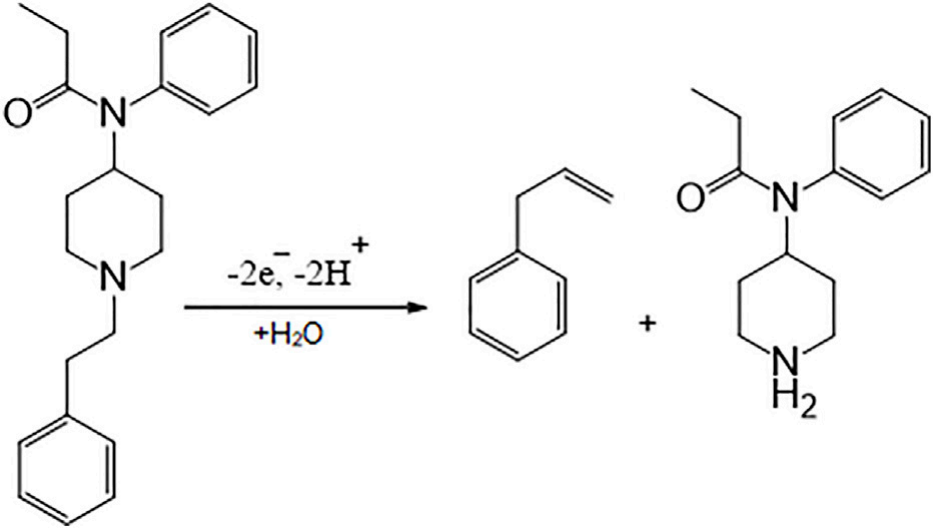
Probable oxidation mechanism for fentanyl.

Concerning the slope of 0.0493 V/pH for fentanyl, they were close to the Nernstian value predicted for an equal number of proton and electron electrochemical process (Scheme 1) (Bard and Faulkner, 2001).

### 3.5 The effect of the scan rate on electrochemical behaviors of fentanyl

**FIGURE 9:**
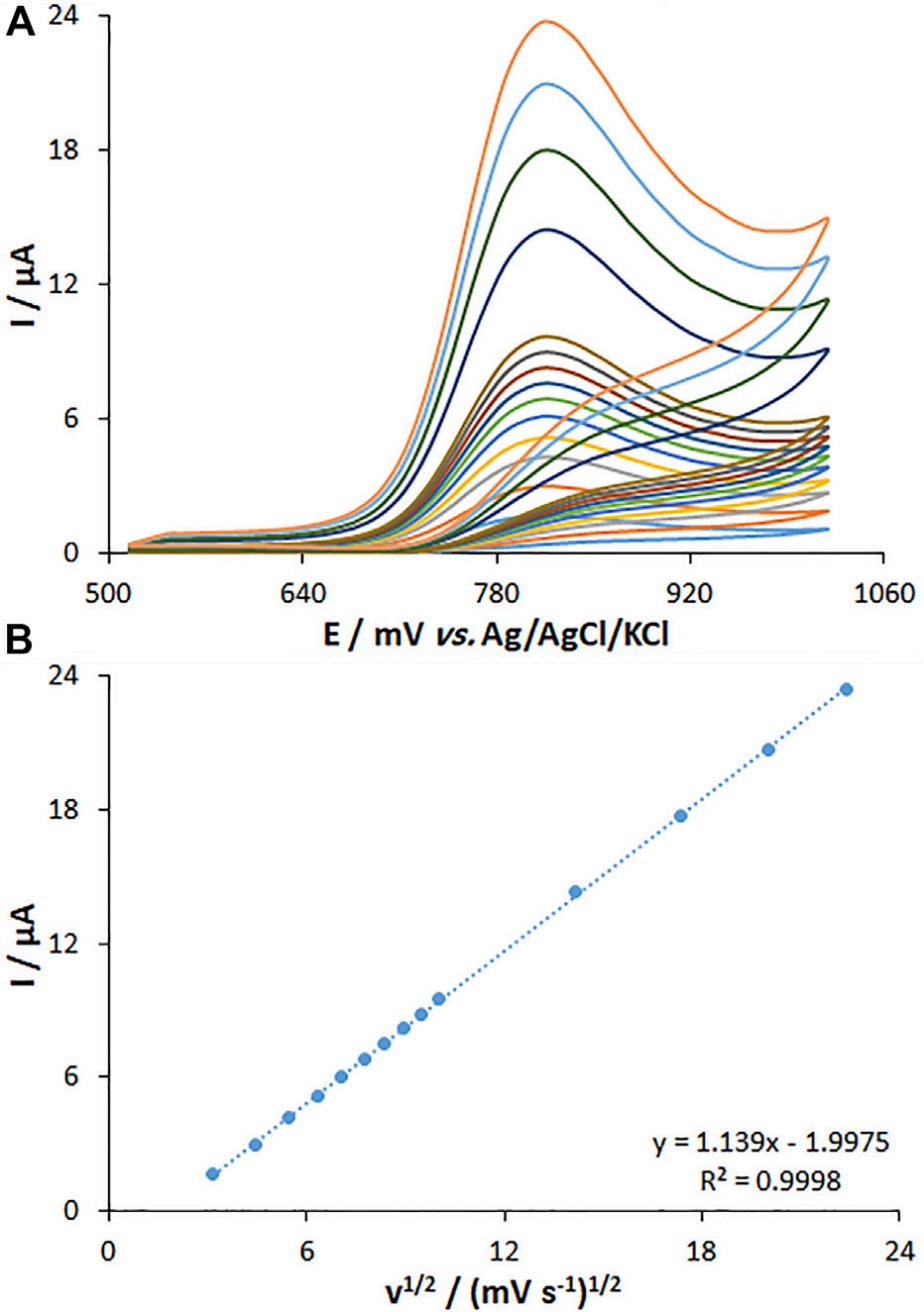
**(A)** CVs of MWCNT/HG-NiO-ND/PGE at pH 7.0 in the presence of fentanyl (20.0 µM) at various scan rates (from inner to outer curves): 10, 20, 30, 40, 50, 60, 70, 80, 90, 100, 200, 300, 400, and 500 mV/s. **(B)** Plots of peak currents vs. υ^1/2^.

The effect of the scan rate on the peak current of fentanyl oxidation was explored by the CV method on the MWCNT/HG-NiO-ND/PGE. The peak current intensity was elevated with the increasing scan rate, as shown in Figure 9A. Figure 9B highlights the current fit with the scan rate square root (10–500 mV/s), which means the redox reactions are controlled by fentanyl diffusion. Data suggested that the scan rate of 50 mV/s was the best for peak currents and peak separation.

### 3.6. The chronoamperometric analysis

**FIGURE 10:**
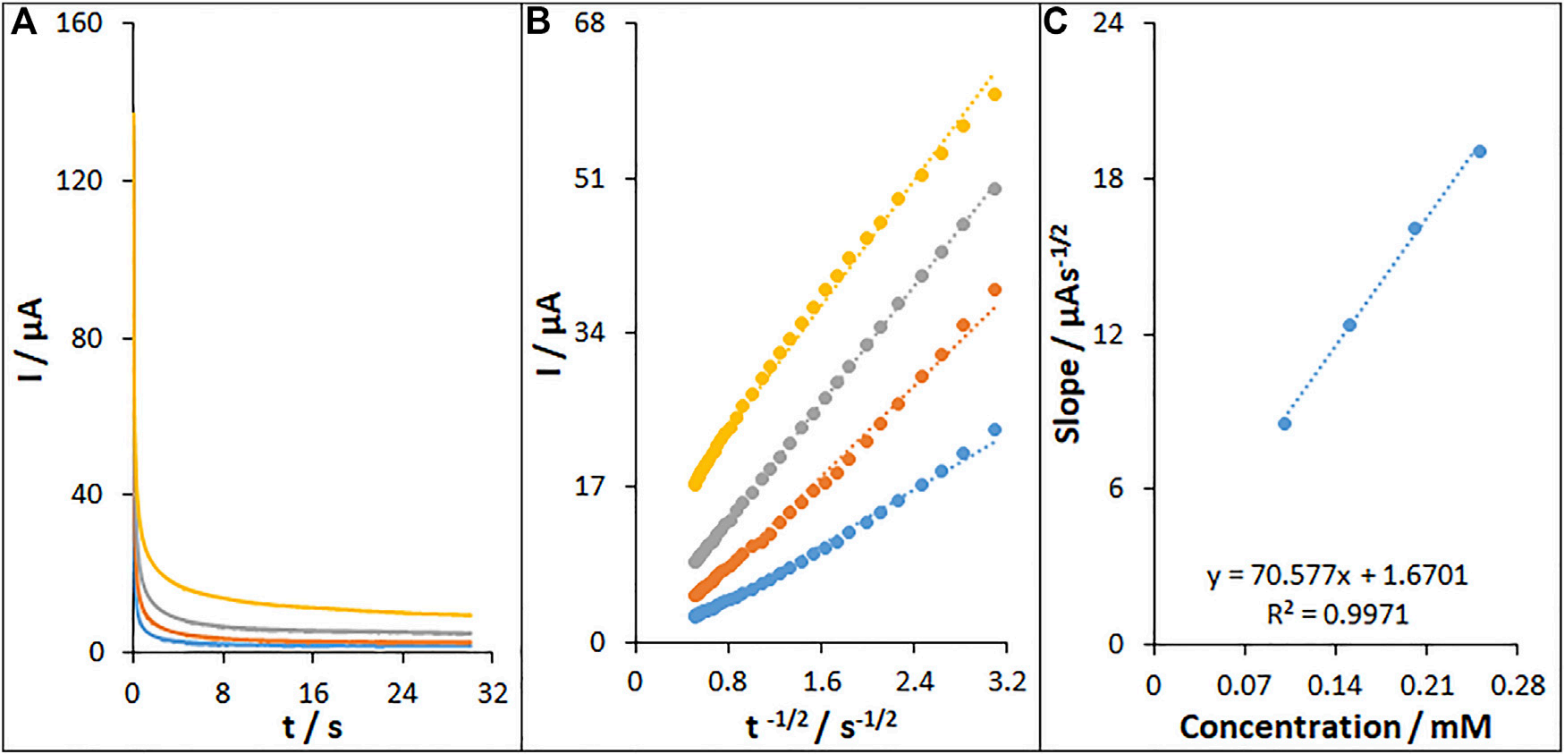
**(A)** Chronoamperograms of fentanyl on MWCNT/HG-NiO-ND/PGE in 0.1 M PBS (pH 7.0) for different concentrations of fentanyl. The numbers 1–4 correspond to 0.1, 0.15, 0.2, and 0.25 mM of fentanyl, respectively. **(B)** Plots of I vs. t^−1/2^ obtained from chronoamperograms 1–4. **(C)** Plot of the slope of the straight lines against fentanyl concentration.

Figure 10A shows the chronoamperometric determinations of fentanyl on the MWCNT/HG-NiO-ND/PGE at a potential of 871 mV for variable fentanyl contents in PBS at pH 7. For the fentanyl with a determined diffusion coefficient (D), the Cottrell equation refers to the electrochemical reaction current with a mass transport-limited rate (Bard and Faulkner, 2001).

$$I=nFAD^{1/2}C_{b}\pi^{-1/2}t^{-1/2}.$$ (4)

Figure 10B illustrates a linear plot of I versus t^−1/2^ under the diffusion-controlled process; the D value can be obtained for fentanyl based on the linear part of the slope of the Cottrell plot, as shown in Figure 10C, which was 4.1 × 10^–6^ cm^2^/s.

### 3.7 The determination of fentanyl

**FIGURE 11:**
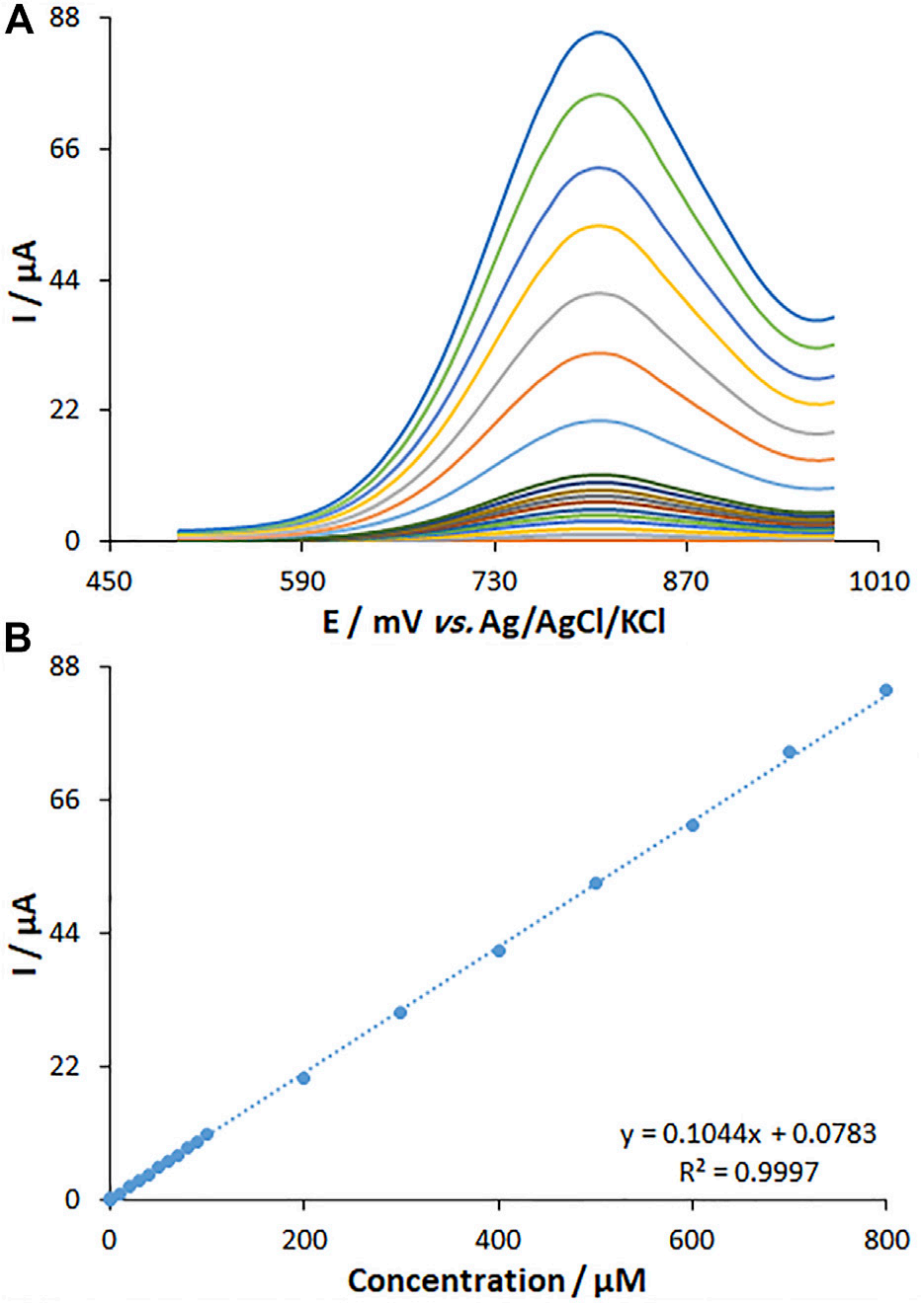
**(A)** DPVs of fentanyl on MWCNT/HG-NiO-ND/PGE in PBS (pH 7.0) at the scan rate of 50 mV s^−1^. Concentrations of fentanyl from inner to outer curves: 0.01, 1.0, 10.0, 20.0, 30.0, 40.0, 50.0, 60.0, 70.0, 80.0, 90.0, 100.0, 200.0, 300.0, 400.0, 500.0, 600.0, 700.0, and 800.0 (μM). **(B)** Plots of I vs. concentrations.

The DPV method was utilized to clarify the relationship between the peak current and fentanyl content (Figure 11). The DPV curves show the oxidation peaks. The peak currents of fentanyl oxidation on the surface of MWCNT/HG-NiO-ND/PGE were linearly related to variable fentanyl contents (0.01–800.0 μM), and the LOD value was as low as 6.7 nM.

The fentanyl content was determined by seven consecutive measurements (150.0 μM), and the relative standard deviation (RSD%) was obtained to be 1.8, which means high efficiency of the proposed protocol for the fentanyl determination.

### 3.8 The interference analysis

The possible interferences of other analytes were explored by DPV in the fentanyl determination. According to the results, there was no significant interference from both diverse anions and cations (Na^+^, Ca^2+^, K^+^, Zn^2+^, Ni^2+^, NO^2-^, Cl^−^, and SO_4_^2-^) and l-phenylalanine, leucine, alanine, uric acid, l-glutamic acid, l-tryptophan, ascorbic acid, l-tyrosine, and epinephrine at 150.0 M concentration with the signal deviations of less than 5%.

### 3.9 The analysis of real specimens

The practical potential of the proposed fentanyl sensor was explored in the drug tablets and blood serum specimens. The reliability of the method was tested by sensing the fentanyl in the specimens spiked with certain content of the analyte (Table 1). The as-fabricated fentanyl sensor applicability was confirmed on the basis of impressive recovery rates obtained between 98.62 and 101.5%.

**TABLE 1:** Determination of fentanyl in fentanyl tablets and human blood serum samples. All concentrations are given in μM (*n* = 5).

Sample	Spiked	Found	Recovery (%)
Fentanyl tablets	0	5.3 ± 3.4	—
	10.0	15.1 ± 2.6	98.69
Human blood serum	0	—	—
	20.0	20.3 ± 2.4	101.5

### 3.10 Reproducibility and stability

Repeatability and stability of the electrochemically as-fabricated fentanyl sensor were tested by the DPV method under the optimized circumstances. Six successive applications of MWCNT/HG-NiO-ND/PGE to quantify 150.0 μM of fentanyl showed no clear alteration in the DPV response. The RSD value of 0.94% validated the commendable repeatability of our sensor. To determine the sensor stability, it was incubated at room temperature for three consecutive weeks, the results of which revealed no distinct change in the peak current (2.82%), which means long-lasting stability of our electrode under the optimized circumstances.

### 3.11 Comparison of our method with others in the literature

The comparison of analytical efficacy between the as-fabricated electrode and other electrochemical methods was performed for fentanyl (Tables). Based on Table 2, the performance of our proposed electrochemical electrode for sensing fentanyl displayed a comparable linear range and better detection limit and sensitivity when compared with the other methods (Najafi and Sohuli, 2018; Naghian et al., 2020; Sohouli et al., 2020; Wester et al., 2020). Accordingly, the as-fabricated sensor is potentially able to determine the trace amounts of studied drugs in various media. Moreover, the electrode used for the sensor fabrication is a PGE that has various advantages, like cost-effectiveness, facile modification, admirable accessibility, and lower background current, when compared with other electrodes.

**TABLE 2:** Performance comparison of MWCNT/HG-NiO-ND/PGE for the determination of fentanyl with other electroanalytical methods.

Modifier	Linear range	Detection limit	Reference
Single-walled carbon nanotube	0.01–1.0 μM	11.0 nM	Wester et al. (2020)
Zinc-based metal–organic framework	1.0–100.0 μM	0.3 μM	Naghian et al. (2020)
Carbon nano-onions	1.0–60.0 μM	300.0 nM	Sohouli et al. (2020)
Multiwall carbon nanotubes and Fe_2_O_3_ nanoparticles	0.08–100.0 nM	45.0 nM	Najafi and Sohuli (2018)
MWCNT and HG-NiO-ND	0.01–800.0 μM	6.7 nM	This work

## 4 Conclusion

This study introduces a new fentanyl electrochemical sensor based on carbon nanotubes combined with HG-NiO-NDs. The production of HG-NiO-NDs added merits and was considered a green approach in the construction of non-toxic sensing systems. The electrochemical behavior of the modified electrode was evaluated for the electrocatalytic oxidation of fentanyl under optimal conditions of electrolytes (0.1 M PBS, pH = 7; a scan rate of 50 mV/s). It was found that the modified electrode could sense fentanyl in the linear range of 0.01 × 10^–6^ to 800.0 × 10^–6^ M. These research findings demonstrated the synergetic impact of MWCNTs and HG-NiO-NDs on the oxidation of fentanyl *via* declining oxidation over-potential and enhancing the oxidation peak current. Reproducible responses together with a lower limit of detection (6.7 nM), in comparison with other literature works, can be attained through the utility of this electrode. The modified electrode was able to provide a very sensitive and stable behavior to fentanyl determination without interference under optimal conditions. In addition, sensitiveness and functionality of the suggested electrochemical sensor toward fentanyl were explored by analyzing pharmaceutical samples, which was accompanied by acceptable outcomes.

## Data Availability

The original contributions presented in the study are included in the article/Supplementary Material; further inquiries can be directed to the corresponding author.

## References

[B1] Ahmad N. Alam M. Wahab R. Ahmad J. Ubaidullah M. Ansari A. A. (2019). Synthesis of NiO–CeO_2_ nanocomposite for electrochemical sensing of perilous 4-nitrophenol. J. Mat. Sci. Mat. Electron. 30, 17643–17653. 10.1007/s10854-019-02113-2

[B2] Al-Enizi A. M. Ahmed J. Ubaidullah M. Shaikh S. F. Ahamad T. Naushad M. (2020). Utilization of waste polyethylene terephthalate bottles to develop metal-organic frameworks for energy applications: A clean and feasible approach. J. Clean. Prod. 248, 119251. 10.1016/j.jclepro.2019.119251

[B3] Al-Enizi A. M. Ubaidullah M. Ahmed J. Ahamad T. Ahmad T. Shaikh S. F. (2020). Synthesis of NiOx@NPC composite for high-performance supercapacitor via waste PET plastic-derived Ni-MOF. Compos. Part B Eng. 183, 107655. 10.1016/j.compositesb.2019.107655

[B4] Al-Enizi A. M. Ubaidullah M. Kumar D. (2021). Carbon quantum dots (CQDs)/Ce doped NiO nanocomposite for high performance supercapacitor. Mat. Today Commun. 27, 102340. 10.1016/j.mtcomm.2021.102340

[B5] Antherjanam S. Saraswathyamma B. (2022). Simultaneous electrochemical determination of hydrazine and hydroxylamine on a thiadiazole derivative modified pencil graphite electrode. Mat. Chem. Phys. 275, 125223. 10.1016/j.matchemphys.2021.125223

[B6] Bard A. J. Faulkner L. R. (2001). Electrochemical methods: Fundamentals and applications. second ed. New York: Wiley.

[B7] Dalkiran B. Brett C. M. A. (2022). Poly(safranine T)-deep eutectic solvent/copper oxide nanoparticle-carbon nanotube nanocomposite modified electrode and its application to the simultaneous determination of hydroquinone and catechol. Microchim. J. 179, 107531. 10.1016/j.microc.2022.107531

[B8] Ebrahimzadeh H. Yamini Y. Gholizade A. Sedighi A. Kasraee S. (2008). Determination of fentanyl in biological and water samples using single-drop liquid–liquid–liquid microextraction coupled with high-performance liquid chromatography. Anal. Chim. Acta X. 626, 193–199. 10.1016/j.aca.2008.07.047 18790121

[B9] Farvardin N. Jahani Sh. Kazemipour M. Foroughi M. M. (2020). The synthesis and characterization of 3D mesoporous CeO_2_ hollow spheres as a modifier for the simultaneous determination of amlodipine, hydrochlorothiazide and valsartan. Anal. Methods 12, 1767–1778. 10.1039/d0ay00022a

[B10] Fathi Z. Jahani Sh. Shahidi Zandi M. Foroughi M. M. (2020). Synthesis of bifunctional cabbage flower–like Ho^3+^/NiO nanostructures as a modifier for simultaneous determination of methotrexate and carbamazepine. Anal. Bioanal. Chem. 412, 1011–1024. 10.1007/s00216-019-02326-8 31897563

[B11] Foroughi M. M. Jahani Sh. Aramesh-Boroujeni Z. Vakili Fathabadi M. Hashemipour Rafsanjani H. Rostaminasab Dolatabad M. (2021). Template-free synthesis of ZnO/Fe_3_O_4_/Carbon magnetic nanocomposite: Nanotubes with hexagonal cross sections and their electrocatalytic property for simultaneous determination of oxymorphone and heroin. Microchem. J. 170, 106679. 10.1016/j.microc.2021.106679

[B12] Foroughi M. M. Jahani Sh. Aramesh-Boroujeni Z. Rostaminasab Dolatabad M. Shahbazkhani K. (2021). Synthesis of 3D cubic of Eu^3+^/Cu_2_O with clover-like faces nanostructures and their application as an electrochemical sensor for determination of antiretroviral drug nevirapine. Ceram. Int. 47, 19727–19736. 10.1016/j.ceramint.2021.03.311

[B13] Foroughi M. M. Jahani Sh. (2022). Investigation of a high-sensitive electrochemical DNA biosensor for determination of Idarubicin and studies of DNA-binding properties. Microchem. J. 179, 107546. 10.1016/j.microc.2022.107546

[B14] Foroughi M. M. Noroozifar M. Khorasani-Motlagh M. (2015). Simultaneous determination of hydroquinone and catechol using a modified glassy carbon electrode by ruthenium red/carbon nanotube. J. Iran. Chem. Soc. 12, 1139–1147. 10.1007/s13738-014-0575-7

[B15] Foroughi M. M. Ranjbar M. (2017). Microwave-assisted synthesis and characterization photoluminescence properties: A fast, efficient route to produce ZnO/GrO nanocrystalline. J. Mat. Sci. Mat. Electron. 28, 1359–1363. 10.1007/s10854-016-5668-x

[B16] Haddad A. Comanescu M. A. Green O. Kubic T. A. Lombardi J. R. (2018). Detection and quantitation of trace fentanyl in heroin by surface-enhanced Raman spectroscopy. Anal. Chem. 90, 12678–12685. 10.1021/acs.analchem.8b02909 30247896

[B17] Haunsbhavi K. Arun Kumar K. D. Ubaidullah M. Shaikh S. F. Venkatesh R. Alagarasan D. (2022). The effect of rare-earth element (Gd, Nd, La) doping of NiO films on UV photodetector. Phys. Scr. 97, 055815. 10.1088/1402-4896/ac64d4

[B18] Ichiyanagia Y. Wakabayashia N. Yamazaki J. (2003). Magnetic properties of NiO nanoparticles. Phys. B Condens. Matter 329, 862–863. 10.1016/s0921-4526(02)02578-4

[B19] Jahani Sh. (2018). Evaluation of the usefulness of an electrochemical sensor in detecting ascorbic acid using a graphite screen-printed electrode modified with NiFe_2_O_4_ nanoparticles. Anal. Bioanal. Electrochem. 10, 739–750.

[B20] Jahani Sh. Sedighi A. Toolabi A. Foroughi M. M. (2022). Development and characterization of La_2_O_3_ nanoparticles@snowflake-like Cu_2_S nanostructure composite modified electrode and application for simultaneous detection of catechol, hydroquinone and resorcinol as an electrochemical sensor. Electrochim. Acta 416, 140261. 10.1016/j.electacta.2022.140261

[B21] Khand A. A. Lakho S. A. Tahira A. Ubaidullah M. Alothman A. A. Aljadoa K. (2021). Facile electrochemical determination of methotrexate (MTX) using glassy carbon electrode-modified with electronically disordered NiO nanostructures. Nanomaterials 11, 1266. 10.3390/nano11051266 34065856 PMC8150394

[B22] Li Q. Wang L. S. Hu B. Y. Yang C. Zhou L. Zhang L. (2007). Preparation and characterization of NiO nanoparticles through calcination of malate gel. Mat. Lett. 61, 1615–1618. 10.1016/j.matlet.2006.07.113

[B23] Maaref H. Foroughi M. M. Sheikhhosseini E. Akhgar M. R. (2018). Electrocatalytic oxidation of sulfite and its highly sensitive determination on graphite screen printed electrode modified with new schiff base compound. Anal. Bioanal. Electrochem. 10, 1080–1092.

[B24] Moarefdoust M. M. Jahani Sh. Moradalizadeh M. Motaghi M. M. Foroughi M. M. (2021). An electrochemical sensor based on hierarchical nickel oxide nanostructures doped with indium ions for voltammetric simultaneous determination of sunset yellow and tartrazine colorants in soft drink powders. Anal. Methods 13, 2396–2404. 10.1039/d1ay00306b 33982698

[B25] Naghian E. Marzi Khosrowshahi E. Sohouli E. Ahmadi F. Rahimi-Nasrabadi M. Safarifard V. (2020). A new electrochemical sensor for the detection of fentanyl lethal drug by a screen-printed carbon electrode modified with the open-ended channels of Zn(II)-MOF. New J. Chem. 44, 9271–9277. 10.1039/d0nj01322f

[B26] Najafi M. Sohuli S. (2018). Electrochemical sensor for fentanyl determination by modified electrode with carbon nanotube and iron (III) oxide nanoparticles. J. Appl. Res. Chem. 12, 103.

[B27] Saffer C. S. Minkowitz H. S. Ding L. Danesi H. Jones J. B. (2015). Fentanyl iontophoretic transdermal system versus morphine intravenous patient-controlled analgesia for pain management following gynecological surgery: A meta-analysis of randomized, controlled trials. Pain Manag. 5, 339–348. 10.2217/pmt.15.29 26088721

[B28] Salajegheh M. Ansari M. Foroughi M. M. Kazemipour M. (2019). Computational design as a green approach for facile preparation of molecularly imprinted polyarginine-sodium alginate-multiwalled carbon nanotubes composite film on glassy carbon electrode for theophylline sensing. J. Pharm. Biomed. Anal. 162, 215–224. 10.1016/j.jpba.2018.09.032 30265981

[B29] Santana E. R. Martins E. C. Spinelli A. (2021). Electrode modified with nitrogen-doped graphene quantum dots supported in chitosan for triclocarban monitoring. Microchem. J. 167, 106297. 10.1016/j.microc.2021.106297

[B30] Saraji M. Boroujeni M. K. (2011). Analysis of narcotic drugs in biological samples using hollow fiber liquid–phase microextraction and gas chromatography with nitrogen phosphorus detection. Microchim. Acta 174, 159–166. 10.1007/s00604-011-0612-5

[B31] Sohouli E. Keihan A. H. Shahdost-fard F. Naghian E. Plonska-Brzezinska M. E. Rahimi-Nasrabadig M. (2020). A glassy carbon electrode modified with carbon nanoonions for electrochemical determination of fentanyl. Mater. Sci. Eng. C 110, 110684. 10.1016/j.msec.2020.110684 32204112

[B32] Stiller R. L. Scierka A. M. Davis P. J. Cook D. R. (1990). A brief technical communication: Detection of fentanyl in urine. Forensic Sci. Int. 44, 1–6. 10.1016/0379-0738(90)90160-z 2303204

[B33] Ubaidullah M. Ahmed J. Ahamad T. Shaikh S. F. Alshehri S. M. Al-Enizi A. M. (2020). Hydrothermal synthesis of novel nickel oxide@nitrogenous mesoporous carbon nanocomposite using costless smoked cigarette filter for high performance supercapacitor. Mat. Lett. 266, 127492. 10.1016/j.matlet.2020.127492

[B34] Ubaidullah M. Al-Enizi A. M. Shaikh S. Ghanem M. A. Mane R. S. (2020). Waste PET plastic derived ZnO@NMC nanocomposite via MOF-5 construction for hydrogen and oxygen evolution reactions. J. King Saud Univ. - Sci. 32, 2397–2405. 10.1016/j.jksus.2020.03.025

[B35] Vakili Fathabadi M. Hashemipour Rafsanjani H. Foroughi M. M. Jahani Sh. Arefi Nia N. (2020). Synthesis of magnetic ordered mesoporous carbons (OMC) as an electrochemical platform for ultrasensitive and simultaneous detection of thebaine and papaverine. J. Electrochem. Soc. 167, 027509. 10.1149/1945-7111/ab6446

[B36] Vignesh S. Suganthi S. Srinivasan M. Tamilmani A. Sundar J. K. Gedi S. (2022). Investigation of heterojunction between α-Fe_2_O_3_/V_2_O_5_ and g-C_3_N_4_ ternary nanocomposites for upgraded photo-degradation performance of mixed pollutants: Efficient dual Z-scheme mechanism. J. Alloys Compd. 902, 163705. 10.1016/j.jallcom.2022.163705

[B37] Wester N. Mynttinen E. Etula J. Lilius T. Kalso E. Mikladal B. F. (2020). Single-walled carbon nanotube network electrodes for the detection of fentanyl citrate. ACS Appl. Nano Mat. 3, 1203–1212. 10.1021/acsanm.9b01951

[B38] Yang H. M. Tao Q. F. Zhang X. C. Tang A. Ouyang J. (2008). Solid-state synthesis and electrochemical property of SnO_2_/NiO nanomaterials. J. Alloys Compd. 459, 98–102. 10.1016/j.jallcom.2007.04.258

[B39] You H. Chen Z. Yu Q. Zhu W. Chen B. Lv Z. (2021). Preparation of a three-dimensional porous PbO_2_-CNTs composite electrode and study of the degradation behavior of p-nitrophenol. Sep. Purif. Technol. 276, 119406. 10.1016/j.seppur.2021.119406

[B40] Zhou Q. Umar A. Sodki E. M. Amine A. Xu L. Gui Y. (2018). Fabrication and characterization of highly sensitive and selective sensors based on porous NiO nanodisks. Sensors Actuators B Chem. 259, 604–615. 10.1016/j.snb.2017.12.050

[B41] Zhou Y. Cheng X. Tynan B. Shao Z. Huang F. Islam M. S. (2021). High-performance hierarchical MnO_2_/CNT electrode for multifunctional supercapacitors. Carbon 184, 504–513. 10.1016/j.carbon.2021.08.051

